# Crossed pulmonary arteries associated with single atrium in an adult: a case report

**DOI:** 10.1186/s12872-016-0354-8

**Published:** 2016-09-05

**Authors:** Fengli Fu, Jiahu Yang, Jianjun Zhang, Yue Feng

**Affiliations:** Department of Radiology, Zhejiang Hospital, No.12 Lingyin Rd, Hangzhou, 310013 China

**Keywords:** Malposition of pulmonary arteries, Crossed pulmonary arteries, Single atrium, Computed tomography

## Abstract

**Background:**

Crossed pulmonary arteries or single atrium is a rare form of cardiovascular anomaly. In previous studies, the anomalies are detected in infant or early adolescence, and infrequently seen in adult population.

**Case presentation:**

We presented a case of the coexistence of two congenital anomalies in a 44-year-old woman who remained well tolerated and undiscovered until adulthood. Physical examination showed a grade III systolic murmur at the cardiac apex, and a grade II/III systolic murmur at left 2–3 intercostal space. An echocardiography revealed absence of atrial septal tissue. Dual-source CT angiography was performed for further evaluation of the great vessel. Except an enlarged single atrium, the imaging showed that the origination of the left pulmonary artery from the pulmonary trunk was superior to that of the right pulmonary artery. The branch pulmonary arteries then crisscrossed as they coursed to their respective lungs. The findings were illustrated by the right heart catheterization and then confirmed at surgery.

**Conclusions:**

To our knowledge, this is the first case report of crossed pulmonary arteries with single atrium as the only additional cardiac anomaly in an adult. Knowledge of this rare combination will be helpful in the differential diagnosis of congenital heart disease and assist the surgeon in treatment planning.

## Background

Crossed pulmonary arteries are a rare form of pulmonary arterial malposition, characterized by an abnormal ostium of the left pulmonary artery (LPA) that originates superior to the right pulmonary artery (RPA) and to its right [1, 2]. Single atrium is regarded as the least common variety among the malformations of the atrial septum, and infrequently seen in adult [3]. In this anomaly, atrial septum is complete absence, and often accompanied by malformations of the atrioventricular valves, especially with a cleft in the mitral valve [4]. Crossed pulmonary arteries or single atrium is often associated with other congenital cardiac and extracardiac disease. Majority of patients with crossed pulmonary arteries or single atrium are symptomatic and poorly tolerated during infancy or early adolescence with dyspnea on effort, fatigue respiratory tract infection, cyanosis [1–4]. Very few cases with crossed pulmonary arteries or single atrium have been reported in adult population. In the following case, we present the combination of two rare congenital anomalies in an adult woman.

## Case presentation

A 44-year-old Chinese woman was referred to our hospital with heart murmur of 40 years duration. She reported no cyanosis, chest congestion and anhelation after activities. On auscultation, there were a grade 3 systolic murmur on apex, and a grade 2/3 systolic murmur on left 2–3 intercostal space.

**Fig. 1:**
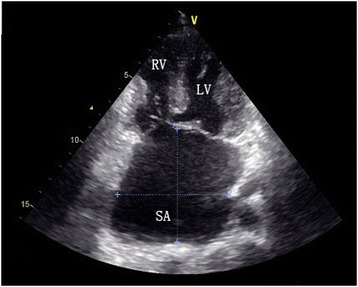
Echocardiography. Echocardiography showed complete absence of interatrial septum. *LV* left ventricle, *RV* right ventricle, *SA* single atrium

Routine laboratory examinations were unremarkable. Chest X-way revealed a mild cardiomegaly and prominent pulmonary trunk. On electrocardiogram, there was normal sinus rhythm, right and left ventricle hypertrophy signs. Echocardiography revealed an absence of atrial septal tissue (Fig. 1) and a small cleft in anterior mitral leaflet. Color doppler examination presented moderate mitral regurgitation and mild tricuspid regurgitation.

**Fig. 2:**
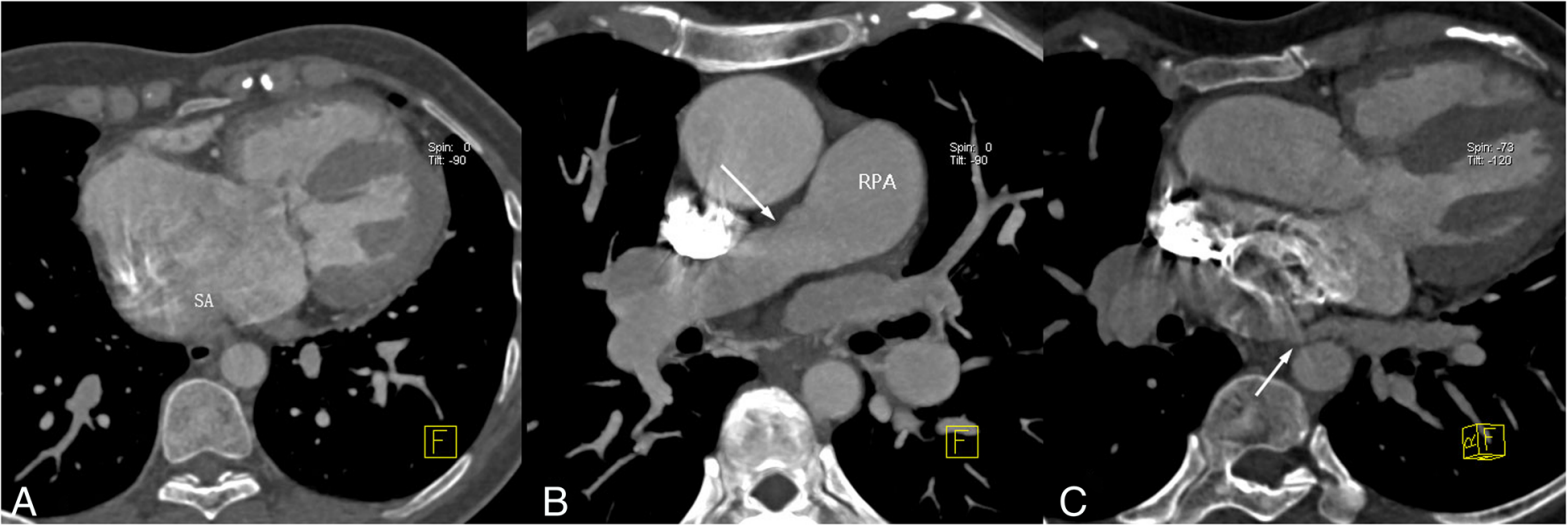
Computed tomography angiography. **a**, **b** Axial CT imaging revealed an enlarged single atrium (SA), and mild stenosis (*arrow*) in the RPA. **c** Axial CTA imaging showed a narrow ostial (*arrow*) by which the left inferior pulmonary vein entered into the left side of a common chamber

**Fig. 3:**
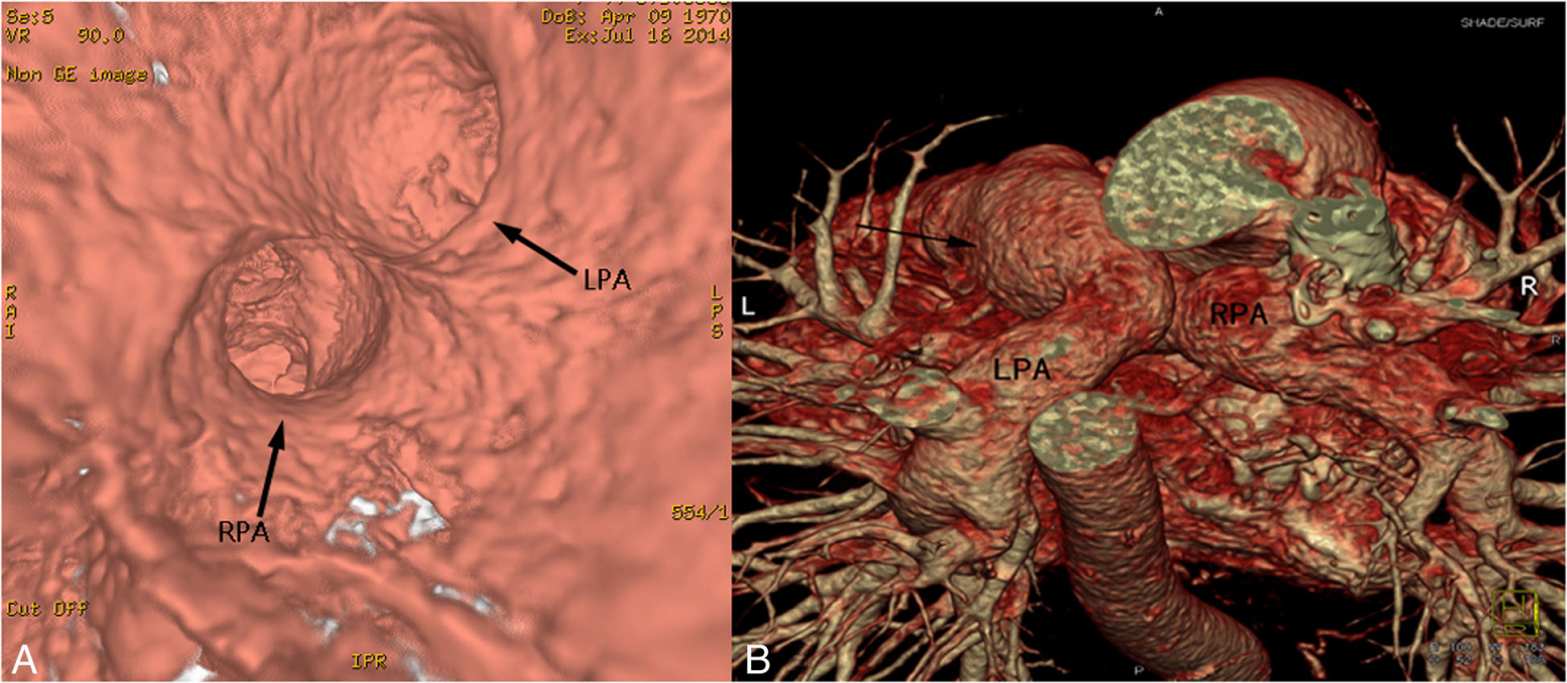
Volume-rendered reconstruction. **a** Virtual endoscopy showed that the origin of the LPA located to the right and superior to the origin of the RPA. **b** Both pulmonary arteries crisscrossed (*arrow*) on course to their respective lungs

**Fig. 4:**
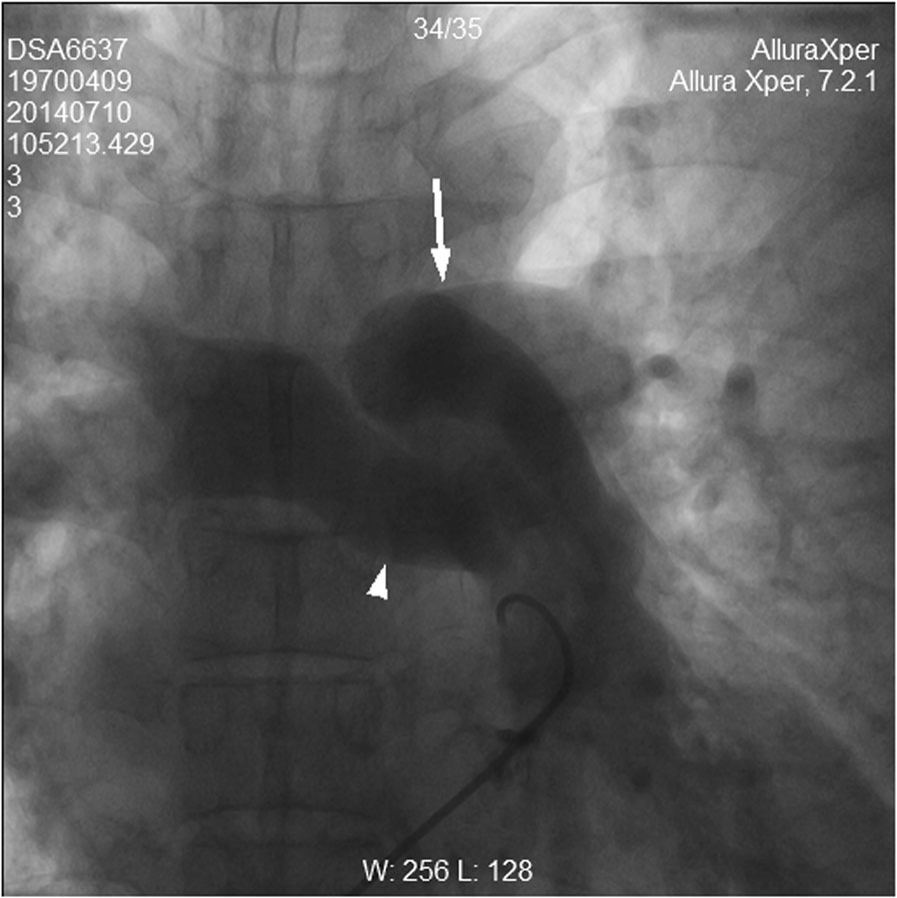
Right heart catheterization. Heart catheterization confirmed the LPA (*arrow*) originated superior to the RPA (*arrowhead*)

Computed tomography angiography (CTA) was performed on a dual-source CT (Somatom Definition, Siemens Medical Systems, Forchheim, Germany) to further evaluate if there were other congenital anomalies of the cardiovascular. It showed an enlarged common chamber (Fig. 2a), and could not characterize any separation between the right and left atria. There was mild stenosis in the right pulmonary artery (Fig. 2b). The left inferior pulmonary vein entered into the left side of a common chamber via a narrow ostial (Fig. 2c). 3D volume rendering image showed the origin of the LPA located to the right and superior to the origin of the RPA (Fig. 3a, b). Both pulmonary arteries crisscrossed on course to their respective lungs (Fig. 3b). The origin and course of the aortic arch, pulmonary trunk and coronary arteries were normal. The right heart catheterization demonstrated the diagnosis of crossed pulmonary arteries and mild stenosis of the right pulmonary artery (Fig. 4).

The patient underwent cardiac surgery to prevent cardiac failure. In operation, there was the absence of atrial septum, a grade 3 cleft in anterior mitral leaflet, absence of cuspis medialis, and crossed pulmonary arteries. The surgical interventions were focused on primary suturing of the cleft on mitral anterior leaflet, tricuspid valvuloplasty, and regeneration of interatrial septum with autologue pericardial patch. Twenty-four months after operation, the patient was in good functional status. Echocardiography showed mild regurgitation in mitral regurgitation; systolic pressure of pulmonary valve was 31 mm Hg.

Crossed pulmonary arteries are a classical form of the malposition of the pulmonary arteries. In this anomaly, the ostium of LPA lies to the right of and above that of the RPA, then the two pulmonary arteries cross one another as they proceed to their respective lungs [1]. Since the first description of crossed pulmonary arteries was provided by Jue et al. in 1966, no more than 69 cases of crossed pulmonary arteries were diagnosed by echocardiography or three-dimensional computed tomographic imaging [1]. The prevalence of crossed pulmonary arteries is approximately 0.06 % in population who undergo chest CT or CTA [5]. The etiology of this anomaly is uncertain. Jue et al. [1] suggested that crossed pulmonary arteries were derived from faulty differential growth during the partitioning of the truncus arteriosus into the aorta and pulmonary trunk, resulting in counterclockwise rotation of the normal origins of the branch pulmonary arteries.

Because of associated underlying congenital heart disease, most patients with crossed pulmonary arteries were symptomatic and discovered in their infancy or early adolescence. According to our search of the medical literature, only one case with crossed pulmonary arteries in adult have been reported, which was diagnosed by echocardiography in a 29-year-old nullipara at routine prenatal [6]. In all reports about the crossed pulmonary arteries, our case is the eldest. The patient remained asymptomatic and relatively well tolerated into adulthood. There was no symptom of fatigue exertional dyspnea or palpitation in patient, except heart murmur. The phenomenon may be further confirms the theory that the hemodynamics of crossed pulmonary arteries is benign, despite the abnormal location and course of the branch pulmonary arteries [2, 7].

Single atrium is a rare variety of interatrial communication. Levy et al. proposed that the term single atrium should be used to denote the condition characterized by (1) complete absence of interatrial septum; (2) absence of malformation of the atrioventricular valves; and (3) absence of interventricular communication [8]. Right sided portion of common chamber has anatomic features of right and left sided portion has anatomic features of left atrium, receives blood from pulmonary vein [4]. This condition is usually symptomatic with dyspnea on effort and cyanosis in the first few years of life, duo to partial mixing of systemic venous blood and pulmonary venous blood in the atrium before inflow into each ventricle [9]. However, systemic oxygen saturation > 90 % are not uncommon. In our case, the patient has no cyanosis, chest congestion and anhelation after activities. The clinical manifestation may be interpreted as preferential streaming of blood across the respective AV valves [9].

In the previously studies, crossed pulmonary arteries was often accompanied by congenital cardiovascular disease such as ventricular septal defect, right aortic arch, interrupted aortic arch and persistent truncus arteriosus, and chromosomal abnormalities such as 18 trisomy syndrome [1, 2, 5]. To our knowledge, crossed pulmonary arteries associated with single atrium as the only additional cardiac anomaly has not been reported so far. Due to high spatial resolution, CTA is useful for visualizing this condition. It can provide accurate angiographic information on the origin, course, and termination of pulmonary arteries and adjacent cardiovascular anomalies non-invasively.

## Conclusions

In summary, we present, to our knowledge, for the first time a case of coexistence of two rare congenital anomalies in the form of crossed pulmonary arteries associated with single atrium in an adult. Diagnostic key point of the case is to reveal the true relationship between the LPA and RPA, and an absence of atrial septal tissue. Knowledge of this rare anomaly will help in the differential diagnosis of congenital heart disease and assist the surgeon in treatment planning.

## Abbreviations

CTA: Computed tomography angiography
LPA: Left pulmonary artery
RPA: Right pulmonary artery
